# Non-allergic Hypersensitivity Reactions to Immunoglobulin Preparations in Antibody Deficiencies: What Role for Anti-IgA IgG and Complement Activation?

**DOI:** 10.1007/s12016-024-09007-0

**Published:** 2024-10-22

**Authors:** Aurore Collet, Diane Pelletier de Chambure, Emmanuelle Moitrot, Gaëlle Breyne, Floriane Mirgot, Stéphanie Rogeau, Mathieu Tronchon, Amélie Nicolas, Sébastien Sanges, Sarah Stabler, Emmanuel Ledoult, Louis Terriou, David Launay, Eric Hachulla, Myriam Labalette, Sylvain Dubucquoi, Guillaume Lefèvre

**Affiliations:** 1 Univ. Lille, U1286 – INFINITE – Institute for Translational Research in Inflammation, Lille, France; 2 INSERM, 59000 Lille, France; 3 CHU Lille, Institut d’Immunologie, Pôle de Biologie Pathologie Génétique, Lille, France; 4 CHU Lille, Service de Pneumologie Et Immuno-Allergologie, Centre de Compétence Maladies Pulmonaires Rares, Lille, France; 5 CHU Lille, Département de Médecine Interne Et Immunologie Clinique, Lille, France; 6 Centre de Référence Des Maladies Auto-Immunes Et Auto-Inflammatoires Systémiques Rares du Nord, Nord-Ouest, Méditerranée Et Guadeloupe (CeRAINOM), Lille, France; 7 Health Care Provider of the European Reference Network On Rare Connective Tissue and Musculoskeletal Diseases Network (ReCONNET), Lille, France; 8 Département de Maladies Infectieuses Et Tropicales, CHU Lille, Lille, France

**Keywords:** Anaphylaxis, Anti-IgA IgG, CARPA, Complement, Intravenous immunoglobulins, Primary immunodeficiency

## Abstract

The presence of IgG anti-IgA in the serum of primary immunodeficiency (PID) patients has long been considered responsible for hypersensitivity (HS) to immunoglobulin preparations (IgPs), but this link is increasingly being questioned. The aim of this work was to describe the prevalence of IgG anti-IgA and its association with HS, and to explore a new pathophysiological hypothesis involving the complement system. We measured IgG anti-IgA, using a standardised commercial technique, in controls and PID patients, and compared our results to a systematic literature review. We measured complement activation in PID patients before and after IgP infusion, and in vitro after incubation of IgP with serum from controls and PID patients. IgG anti-IgA was detected in 6% (*n* = 2/32) of PID patients, 30% (*n* = 3/10) of selective IgA deficiency patients and 2% (*n* = 1/46) of healthy controls. In the literature and our study, 38 PID patients had IgG anti-IgA and HS to IgPs and 9 had IgG anti-IgA but good tolerance to IgPs. In our patients, we observed a constant complement activation after IgP infusion compared to baseline. In vitro, IgP induced significant complement activation with all sera from tested individuals. IgA immunisation is not rare in PID, higher in selective IgA deficiency, but may also occur in healthy controls. Our results question the clinical relevance and pathophysiological implication of IgG anti-IgA in the context of HS with IgPs. Complement activation-related pseudoallergy could explain the clinical characteristics and natural history of HS symptoms.

**Supplementary Information:**

The online version contains supplementary material available at 10.1007/s12016-024-09007-0.

## Introduction

Patients with antibody (Ab) deficiencies can benefit from immunoglobulin replacement therapy (IgRT), which has been used since 1952 [1]. Some patients experience hypersensitivity reactions (HS) to the Ig infusions, which can be severe in some rare cases [2]. In 1962, Gallagher et al*.* described the possibility of immunisation against IgA in IgA-deficient patients (i.e. selective IgA deficiency [sIgA], and common variable immunodeficiency [CVID]), leading to the development of anti-IgA Abs [3]. Thereafter, Vyas et al. associated the risk of HS with the presence of anti-IgA Abs [4]. For this reason, and to mitigate the formation of aggregates in Ig preparations (IgPs) [5], pharmaceutical companies have endeavoured to reduce the amount of IgA, and consequently IgM, in IgPs. Nowadays, conventional IgPs contain a large majority of IgG, with only traces of IgA and IgM.

However, the potential link between anti-IgA antibodies and HS to IgPs is controversial in the literature [6]. Whilst some studies report an increased risk of life-threatening HS in anti-IgA-positive patients [7], others do not retain a clinical significance of these antibodies [8]. Moreover, the mechanisms underlying IgP-induced HS are unclear. The hypothesis of an allergic IgE-mediated mechanism against the IgA contained in the IgPs is unlikely for several reasons. Firstly, assessments for anti-IgA IgE are mostly negative [9–11]. In the rare cases of anti-IgA IgE positivity, it was almost always in association with the presence of anti-IgA IgG [7, 8, 12]. Secondly, this hypothesis contradicts the typical course of IgP-HS, which usually occurs upon the first IgP infusion, without a sensitization period, which is characteristic of allergic HS reactions [13]. Moreover, the symptoms tend to improve with reduced infusion rates and with repeated exposure to IgP (usually at the 2nd infusion, or at the subsequent ones) [13], which does not support an IgE-mediated mechanism.

Conversely, this pattern mirrors another type of HS [14], involving the complement system [15]. This phenomenon, called CARPA (i.e. complement activation-related pseudoallergy), is increasingly recognised as a cause of pseudoallergy symptoms with several drugs, such as liposomal drugs, monoclonal antibodies or pegylated proteins [14]. In addition to the compatible natural history, the hypothesis of a complement activation mechanism for IgP-HS is tempting because Ig aggregates and immune complexes (which can be generated by the IgP infusions) are strong activators of the classical complement pathway [16]. Moreover, the symptoms associated with CARPA are various, not specific (angioedema, bronchospasm, flushing, nausea, chest pain, headache, chills) [14], and fully compatible with those observed in IgP-HS [13, 17] (Table 1).

**Table 1:** CARPA characteristics and compatibility with IgP-HS

	CARPA characteristics Adapted from (30) and (31)	Compatibility with IgP-HS Reviewed in (17)
Reaction kinetics	Reaction at first exposure	Yes
	Reaction is milder or absent upon re-exposure	Yes
	Reaction may be tachyphylactic	Yes
Response to infusion speed	Slowing down the rate of infusion decreases symptoms	Yes
Response to premedication	Steroids, antihistamine	Yes: hydration, antihistamine, NSAID
Reaction rate	High reaction rate (2–10% or higher)	Yes: up to 87%
Symptoms	Flushing, rash, dyspnoea, chest pain, back pain and subjective distress	Yes: flushing, nausea, fatigue, fever, chills, malaise, lethargy, rash, arrhythmia, hypotension

*CARPA*, complement activation related pseudoallergy; *IgP-HS*, hypersensitivity to immunoglobulin preparations; *NSAID*, non-steroidal anti-inflammatory drugs

The aim of this work was to explore the link between anti-IgA IgG and IgP-HS risk, and to unravel the potential implication of the complement system in IgP-HS.

## Methods

### Anti-IgA IgG Assay

We performed anti-IgA IgG assay using a fluorescent enzyme immunoassay (ImmunoCAP™, Thermo Fischer Scientific®) on serum samples, according to the manufacturer’s protocol.

We retrospectively included all the patients who were tested for anti-IgA IgG at Lille University Hospital Centre, France, between March 2022 and January 2024. We systematically collected the following data in the patients’ medical records: presence and type of immune deficiency (CVID, sIgA, specific polysaccharide antibody deficiency, IgG subclass deficiency, combined immunodeficiency, post-rituximab or post-allograft ID), IgA levels, anti-IgA IgG levels, presence or absence of IgRT, and, if applicable, IgRT administration route, type of IgP, IgP-HS presence or absence, and, if present, type of reaction, number of Ig courses before the HS reaction, delay of the reaction since the beginning of the infusion, and tolerance to subsequent Ig infusions.

Serum samples from healthy controls from a cohort of healthcare workers at Lille University Hospital Centre, France, were also tested for anti-IgA IgG.

We also assessed anti-IgA IgG levels in different IgPs.

### Systematic Literature Review on Anti-IgA Antibodies in PIDs

We searched for original articles about anti-IgA Abs and IgP-HS in the Pubmed electronic database. The search was restricted to articles published between 1962 (first description of anti-IgA Ab) and 2023. Various associations of the following keywords were used: anti-IgA antibody, anti-IgA IgG, IgA deficiency, CVID, anaphylaxis, anaphylactoid, infusion reaction, immunoglobulin replacement therapy, prevalence. Relevant articles were selected on their titles; then, abstracts and full texts were reviewed. The literature search was extended using the relevant references of each selected article. We restricted the search to articles reporting reactions to immunoglobulin preparations, and excluded other blood products. Only articles with full text available in English or French were considered (Fig. S1).

### Complement Assays

We first evaluated the in vitro complement activation with IgP thanks to complement split products sC5b9 and Bb by an enzyme-linked immunosorbent assay (Microvue, QuidelOrtho^©^). See the supplementary methods for detailed protocol.

We secondly assessed the in vivo complement activation in patients receiving IgRT, thanks to comparisons of CP50 activity, C3c, C4, complement split products sC5b9 and Bb between baseline and post-IgP infusion. See the Supplementary Methods for details of the protocol.

### Ethics

In line with the regulations laid down by the French National Data Protection Commission (CNIL) and international guidelines, written, informed consent was neither required nor requested for this non-interventional study. In line with French recommendations, the patients’ sample collection was declared to the CNIL (DEC24-089).

The sera from healthy controls consisted of sera previously collected for a study approved by the Ile-De-France V ethics committee (ID‐CRB 2021-A00119-32). All of the healthy subjects had previously been informed about the potential use of the collected samples for future research projects.

## Results

### Anti-IgA IgG in PID Patients and Healthy Controls, Analysed Using an Automated Method

**Fig. 1:**
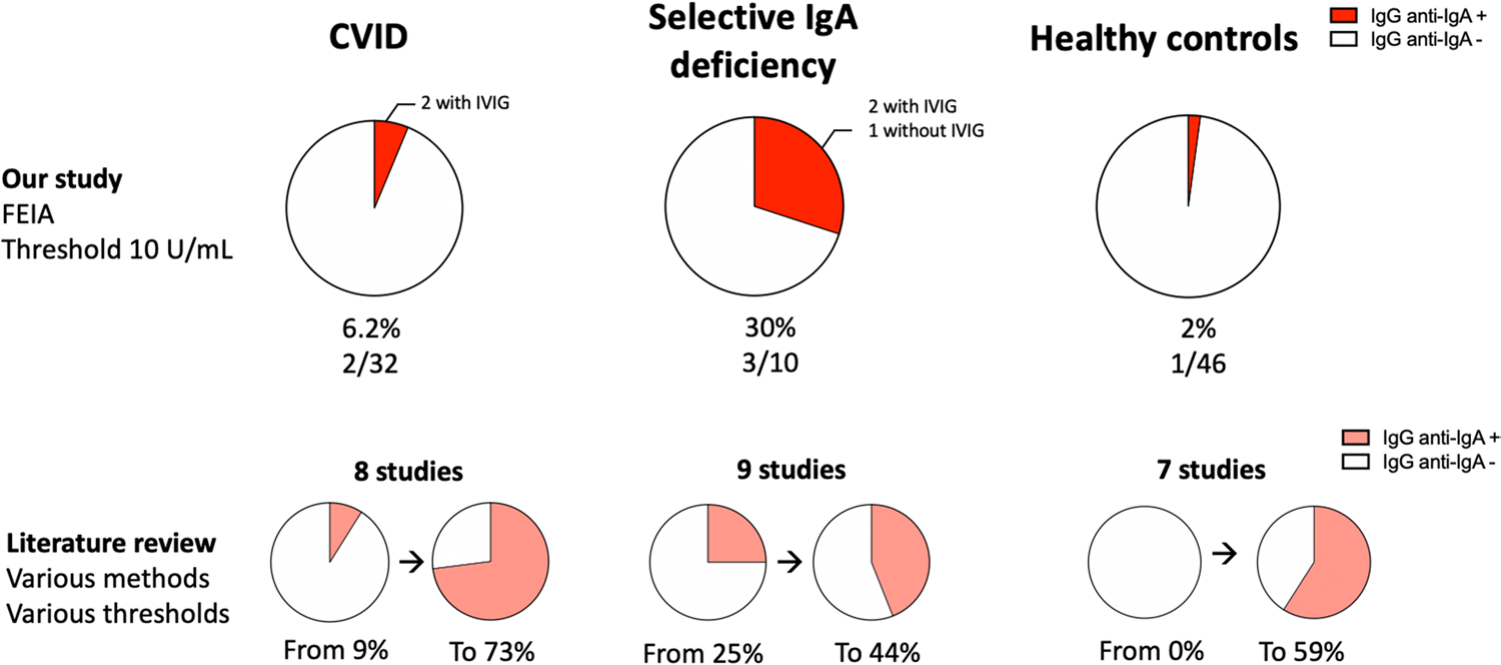
Anti-IgA IgG prevalence in primary immunodeficiency patients and in healthy controls. We performed anti-IgA IgG dosage using a fluorescent enzyme immunoassay on CVID and selective IgA deficiency patients managed at our centre and on 46 healthy controls. The prevalence of anti-IgA IgG in our cohort is presented at the top of the graph. We compared our results with those of studies in the literature which used various anti-IgA IgG assessment methods, and various positivity thresholds. Minimum and maximum prevalence for each group found in the literature are presented at the bottom of the graph. CVID, common variable immunodeficiency; FEIA, fluorescent enzyme immunoassay

We collected the clinical and biological data of all the patients for whom an anti-IgA IgG assay had been performed in our centre. We analysed 152 samples from 131 different patients, using a standardised immunoassay. Of these patients, 90 had a diagnosis of PID. Other patients were explored for autoimmune disorders. Amongst the 131 patients explored, six (4.5%) had positive anti-IgA IgG dosage (Table 2). Anti-IgA IgG was repeatedly searched for in two patients who tested positive and all the repeated samples were also positive (*n* = 5 and *n* = 2 different samples, respectively). Only two of these six patients had previously received IgRT. Overall, in our cohort, two of the 32 CVID patients (6.2%) tested positive (Fig. 1, Table S1), neither of whom had detectable serum IgA, and three of 10 (30%) sIgA patients tested positive (Fig. 1, Table S2). One other patient with isolated IgG3 deficiency had anti-IgA IgG despite normal IgA levels (IgA: 1.21 g/L).

**Table 2:** Description of the anti-IgA IgG-positive patients of the Lille cohort

Gender/age	Main diagnosis	IgA (g/L)	Anti IgA-IgG (U/mL)	IgP	IgP-HS	No. of Ig courses before IgP-HS	HS delay after start of perfusion	Tolerance to the following Ig courses
F/51	sIgA	< 0.07	328	CLAIRYG, < 22 μg/mL, IV	Headache and chills	None	ND	No other infusion
M/46	CVID	< 0.07	131	TEGELINE, 850 μg/mL, IV	Malaise and fever at first perfusion Abdominal pain + throat oedema at 2nd infusion	None	< 5 min	Good tolerance to GAMMAGARD and HIZENTRA
M/53	CVID	< 0.07	232	CLAIRYG, < 22 μg/mL, IV	Chills, throat oedema at 1st infusion	None	ND	Good tolerance to PRIVIGEN
F/19	sIgA	< 0.06	58	No	ND	ND	ND	ND
F/30	sIgA	< 0.06	157	No	ND	ND	ND	ND
M/86	IgG3 deficiency	6.97	33	No	ND	ND	ND	ND

*CVID*, common variable immunodeficiency; *IgP-HS*, hypersensitivity to immunoglobulin preparations; *IgP*, immunoglobulin preparation; *ND*, not determined; *sIgA*, selective IgA deficiency

Amongst 46 healthy subjects (Fig. 1, Table S3), 1 (2%) tested positive for anti-IgA IgG at 46 U/mL (threshold 10 U/mL). No Ig deficiency was identified in this patient (IgG 10.20 g/L, IgA 1.72 g/L, IgM 0.28 g/L).

We also assessed anti-IgA IgG levels in different IgPs, that are composed of Ig derived from the plasma of thousands of healthy donors (Table S4). We found positive anti-IgA IgG levels in 4 amongst the 6 IgP tested when assessed without dilution, and in 2 amongst 6 when diluted to achieve physiological serum IgG levels (10 g/L).

### Anti-IgA IgG in PID Patients and Healthy Controls: Systematic Literature Review

In CVID patients (Fig. 1, Table S1, 8 studies included), anti-IgA immunisation was found to be very heterogeneous, with a prevalence from 9 to 73% of patients. In sIgA (Fig. 1, Table S2, 9 studies included), the results were more homogeneous, with anti-IgA immunisation ranging from 25 to 44% of patients.

Finally, we searched for studies about anti-IgA Abs in healthy individuals. The prevalence of anti-IgA IgG varied widely between studies, from 0 to 59%, with various size samples and various methods of dosage (Fig. 1, Table S3, 7 studies included).

### Clinical Significance of Anti-IgA Abs

After describing anti-IgA Ab prevalence in PID patients and healthy controls, we assessed its clinical significance with regard to IgRT tolerance. We collected the presence or absence of any HS in our local cohort of 54 PID patients treated with IgP and tested for anti-IgA IgG, and we searched the available literature for single case reports and case series focusing on IgP-HS with regard to the patient’s anti-IgA IgG status.

#### Patients with Anti-IgA Abs and IgP-HS: Data from Our Cohort and from a Systematic Literature Search

**Fig. 2:**
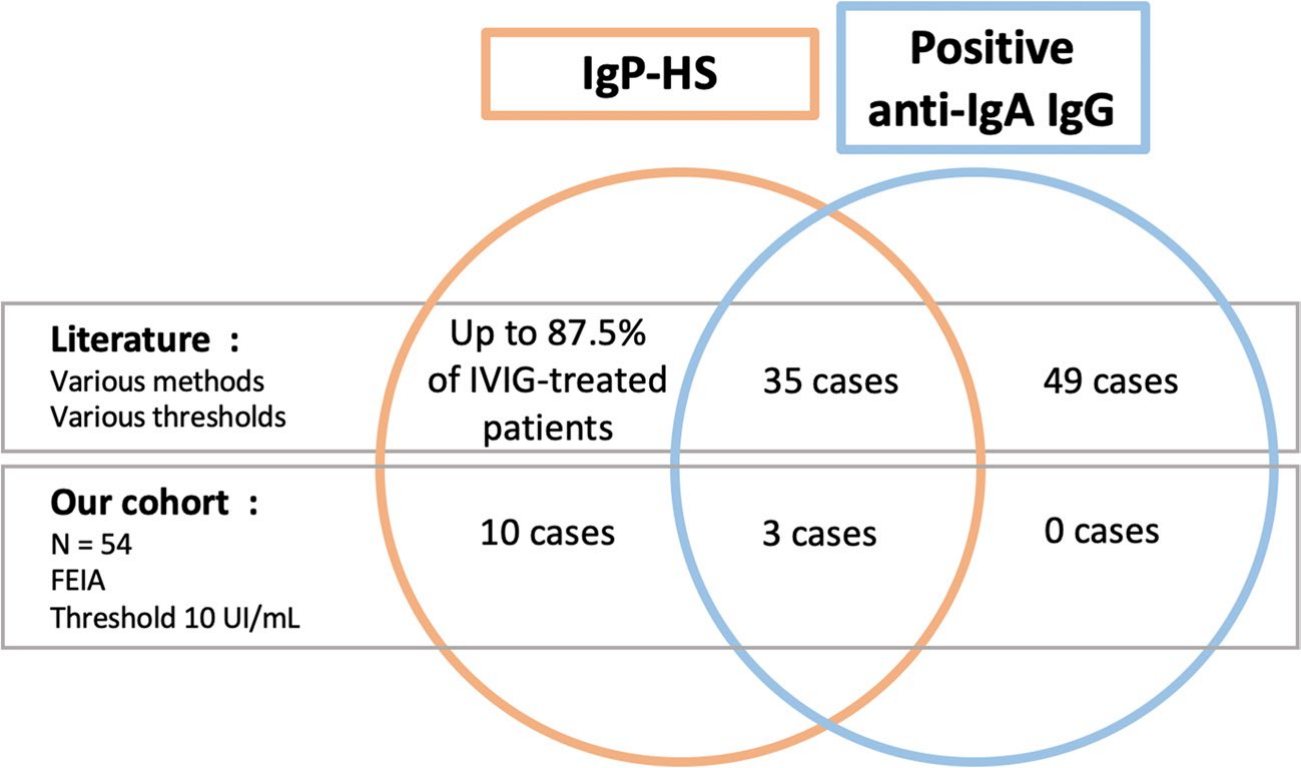
Association between IgP-HS and anti-IgA IgG presence. We studied all the patients who were tested for anti-IgA IgG in our laboratory and systematically collected the presence of IgP-HS in the medical records for each patient. We also performed a literature review about anti-IgA Ab and IgP-HS in the Pubmed electronic database. We report here the number or percentage of patients with (1) IgP-HS without anti-IgA IgG (left), (2) with IgP-HS and anti-IgA IgG (middle), and (3) with anti-IgA IgG without IgP-HS (right). FEIA, fluorescent enzyme immunoassay; IgP-HS, hypersensitivity to immunoglobulin preparations

We identified 38 patients (including 3 of our cohort) with anti-IgA Abs and IgP-HS (Fig. 2, Table 2, Table S5). The majority had a CVID diagnosis (*n* = 34, 89%), three had undefined hypogammaglobulinaemia and one had sIgA (with secondary IgG deficiency due to protein loss enteropathy). All except one had undetectable IgA levels (from < 0.1 to < 0.0009 g/L depending of the assay sensibility). Anti-IgA Ab levels could not be averaged because of the different assay techniques. When it was assessed, the isotype of the anti-IgA antibody was isolated IgG in 92% of cases. One patient had combined anti-IgA IgG and anti-IgA IgE, and one patient had isolated anti-IgA IgE. Various IgPs were responsible for the HS, with very wide range of IgA concentrations (from < 10 to 2500 μg/mL). The administration route was mostly intravenous (IV). The HS reactions were heterogeneous, ranging from mild symptoms (headache, nausea, chills) to severe reactions. The symptoms could appear from the first course of the treatment or after a large number of IgP infusions (up to 24). When described, the delay to symptom onset was usually short, within a few seconds or minutes after initiating the infusion. Despite the HS to an IgP, some patients continued IgRT with the same IgP (at a reduced infusion rate) [10, 18] or with another IgP (mostly IgSC, or sometimes so-called IgA-depleted products) [19, 20], in most cases with an improved level of tolerance. Several authors reported a desensitisation phenomenon: the risk and intensity of adverse reactions decreasing upon repeated exposure to the IgP [13, 18, 20–22].

Regarding the anti-IgA IgG-positive patients in our cohort who received IgP, all experienced at least one episode of HS (Fig. 2, Table 2). Two patients had CVID and one had a primary sIgA and secondary IgG deficiency (protein loss enteropathy). All of them had undetectable IgA levels. HS reactions occurred during the first IgP infusion in all cases. For the 2 patients who received further IgP infusions with another preparation, the therapy was well tolerated.

#### Patients with Anti-IgA Abs and Without IgP-HS

Even if such patients were non-exhaustively described in the literature, we also identified 49 IgP-treated patients with positive anti-IgA Abs, and without IgP-HS (Fig. 2, Table S6): 35 had CVID, seven had sIgA, two had undefined hypogammaglobulinaemia, two had hyper-IgM syndrome and one had combined IgG2/IgA deficiency (missing data: *n* = 2). Their IgA levels ranged between < 0.001 and 0.97 g/L. When assessed, the isotype of the anti-IgA Ab was IgG in most cases, and one patient had combined anti-IgA IgG and anti-IgA IgE. The route of administration could be IV, intramuscular (IM) or subcutaneous (SC).

#### Patients with IgP-HS Without Anti-IgA Ab

After identifying patients who had anti-IgA Abs with or without IgP-HS, we conducted our own study to identify patients who had IgP-HS, and in whom no anti-IgA IgG was detected (Fig. 2, Table S7). Amongst the 51 without anti-IgA IgG and receiving IgRT, we found that 10 patients (19%) had experienced HS at least once. These patients could have IgA deficiency (*n* = 7) or not (*n* = 3). The IgPs concerned were various, with IV or SC route, including preparations with a very low content in IgA (e.g. GAMMAGARD®, 2.2 μg/mL). Symptoms typically included skin eruptions and headaches, which could progress to a meningeal syndrome. These symptoms mostly occurred during the first infusion or within a few hours after.

### Analysis of Complement System Activation with Ig Preparations

Finally, we wanted to explore the potential pathophysiological mechanisms to explain IgP-HS, and in particular the CARPA syndrome.

**Fig. 3:**
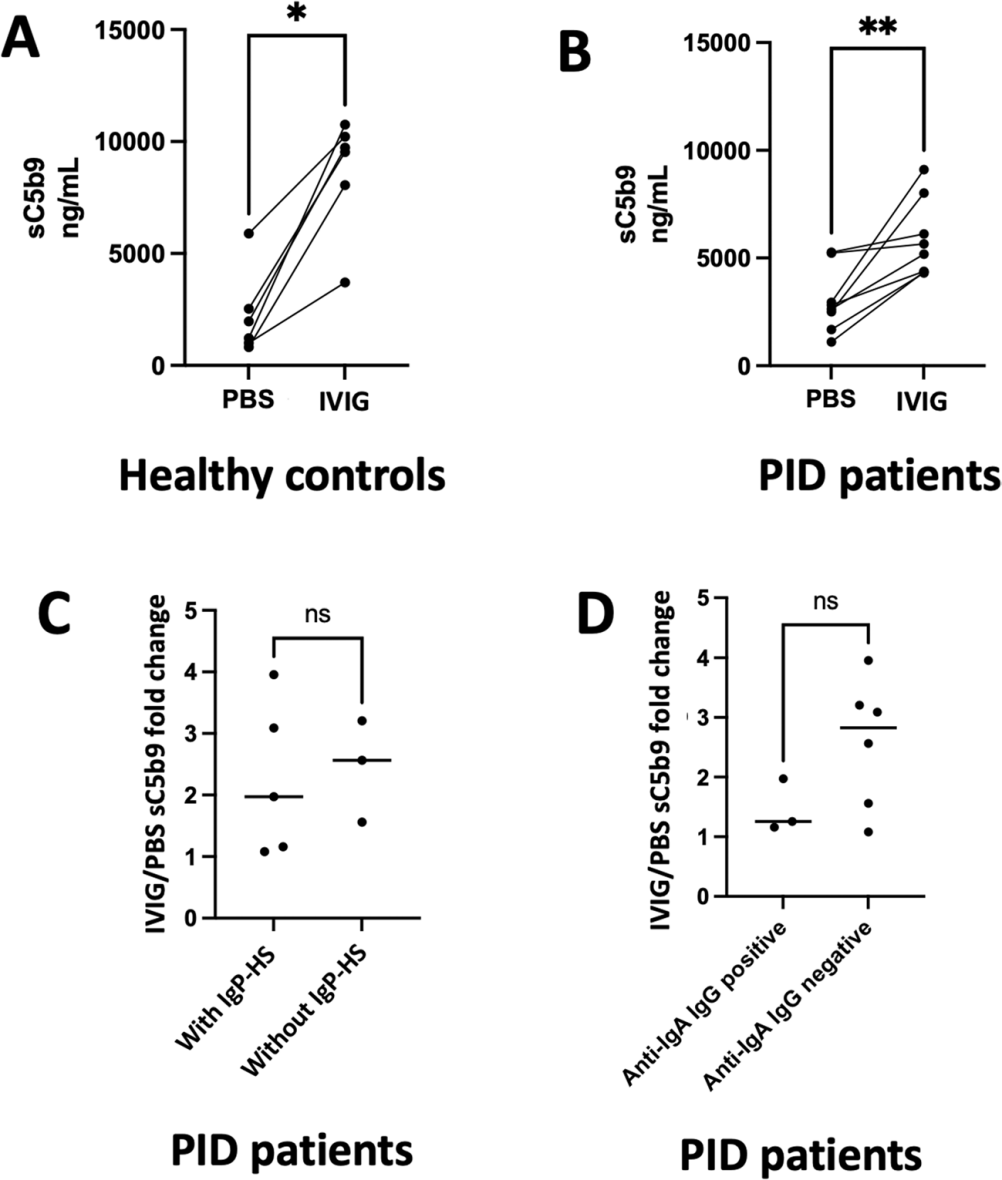
In vitro complement activation tests. Serum samples of healthy controls, anti-IgA-negative and -positive PID patients, and PID patients with or without IgP-HS were incubated with a negative control (PBS) or with an IgP (Clairyg®) for 30 min at 37 °C. The complement split product sC5b9 was dosed to assess the activation of the complement pathway, in healthy controls (**A**) and in PID patients (**B**). A paired Wilcoxon test was used to compare sC5b9 levels after incubation with PBS and with IgP. The increase in sC5b9 levels was assessed after incubation with IgP compared to PBS, according to the presence or absence of a history of HS (**C**) or according to presence or absence of anti-IgA IgG (**D**). Results are presented as fold changes between IgP and PBS experiments (individual values and their median). Mann–Whitney test was used to compare these fold changes in both comparisons, with a significance threshold at 0.05. IgP-HS, hypersensitivity to immunoglobulin preparations; PID, primary immunodeficiency. *p ≤ 0.05; **p ≤ 0.01

To this end, we first tested the propensity of IgPs to activate the complement in vitro, as used for other drugs to assess the CARPA mechanism [31]. Serum samples of healthy controls and of PID patients were incubated at 37 °C with a negative control (PBS) or with an IgP (Clairyg®); then, the complement split product sC5b9 was dosed (Fig. 3). No sC5b9 was detected in 2 different IgPs (data not shown). In all groups (healthy controls, anti-IgA IgG-positive and -negative PID patients, with or without a history of IgP-HS), we observed that sC5b9 constantly increased when serum was incubated with IgP when compared to PBS (mean sC5b9 fold change 2.2 with IgP in comparison to PBS), consistent with a complement activation with IgP. No difference was found between anti-IgA IgG-positive and -negative patients (respectively, 1.26 vs. 2.83 fold change, *p* = 0.27), nor between patients with and patients without a history of IgP-HS (respectively, 1.93 vs. 2.56 fold change, *p* = 0.78).

**Fig. 4:**
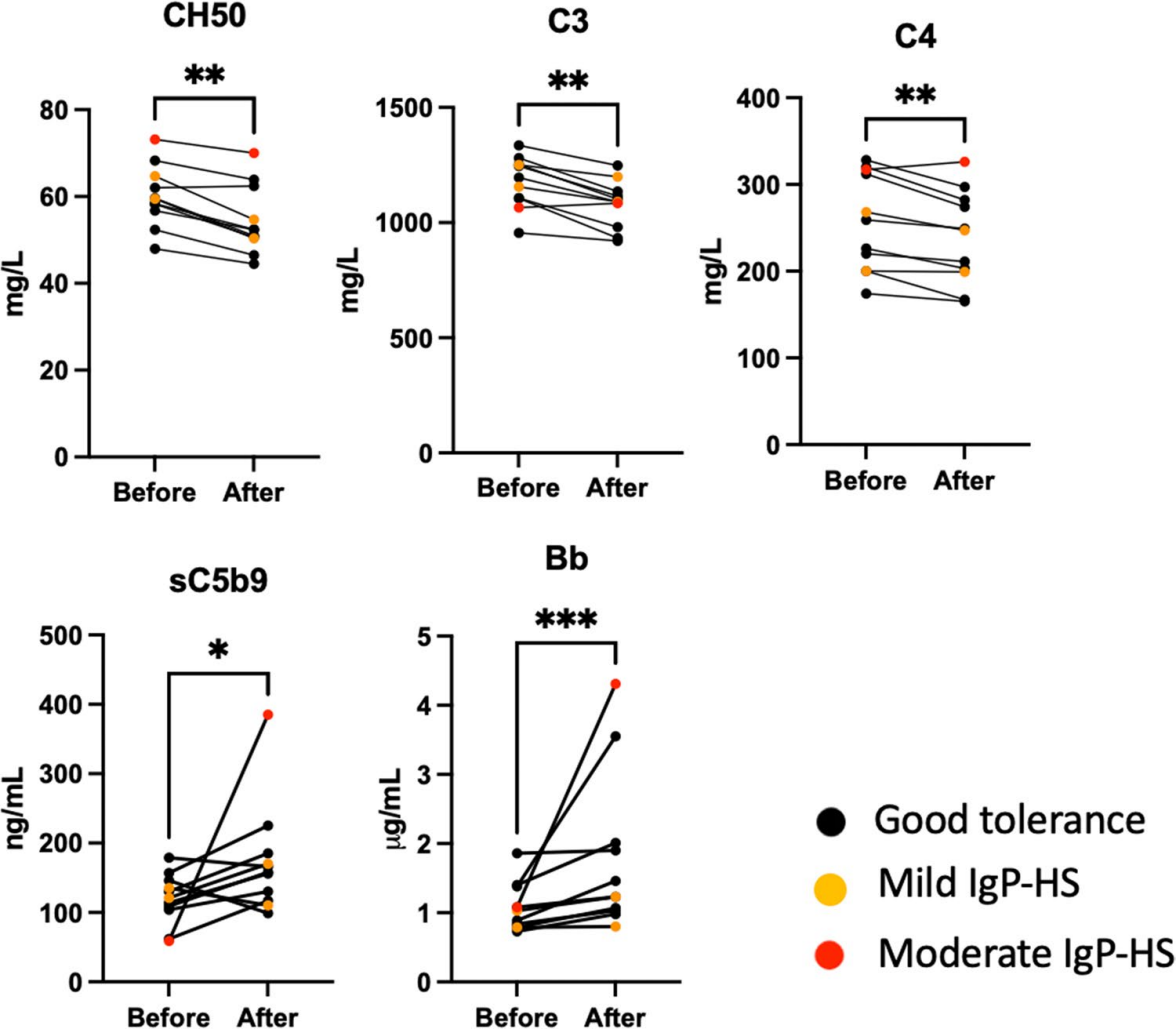
In vivo complement assessment before and after IgP infusion. Plasma samples were collected from 11 PID patients just before and just after an IgIV infusion. Eight patients had no adverse effect (black dots) and three had signs of IgP-HS (mild HS symptoms, orange dots; moderate HS symptoms, red dots). Complement activation was assessed by dosing CH50, C3, C4 and the complement split products sC5b9 and Bb. Individual values before and after the IgP infusion were compared using a paired *t* test. IgP-HS, hypersensitivity to immunoglobulin preparations

We then sought to confirm this complement activation in vivo. We assessed CH50, C3, C4, sC5b9 and Bb just before and after an IgP infusion in 11 PID patients. Three of these patients had IgP-HS, with two mild reactions (headache) and one had a moderate reaction (urticaria) (Fig. 4, Table S8). All tested negative for anti-IgA IgG. No sC5b9 nor Bb was detected in 2 different IgPs (data not shown). In all patients, irrespective of whether or not they had IgP-HS, CH50 decreased and Bb increased after the IgP infusion. In all patients except one (in whom they were stable), C3 and C4 also decreased after IgP. Results were more heterogeneous for sC5b9, with an increase in eight patients and a slight decrease in 3. Overall, the dosages were consistent with complement activation caused by the IgP infusions in all the subjects. This activation did not seem to be higher in the two patients with mild IgP-HS. Remarkably, the patient with moderate IgP-HS had higher levels of sC5b9 and Bb than all the other subjects, consistent with a higher complement activation in vivo.

## Discussion

IgP-HS is a frequent problem in PID patients. Whilst an underlying allergic mechanism, notably IgE- and/or IgG-mediated, has been proposed to account for these hypersensitivity reactions, the evidence supporting this hypothesis is fragile. Our case series and literature review validate the occurrence of IgP-HS even in patients with detectable IgA levels and/or no anti-IgA IgG. Hence, we decided to explore another mechanism, namely complement activation due to massive IgG infusion, considering that IgP-HS symptoms have numerous similarities with CARPA syndrome.

### Our Work First Shows that Anti-IgA IgG Are not Rare in PID Patients, Especially in sIgA, and that Their Occurrence Is not Restricted to IgA-Deficient Individuals

In our centre, which uses a standardised commercial anti-IgA IgG assay, the prevalence of anti-IgA IgG was 6.2% in CVID patients, and 33% in sIgA. Anti-IgA IgG was also found in 2% of healthy controls. In the literature, the percentages varied widely from one study to another. We can formulate several hypotheses to explain these discrepancies between studies, mostly for technical reasons. First, most of the studies are old and used various, non-standardised methods of dosage. The positivity threshold also varied between studies. Furthermore, it should be noted that most of the studies involving healthy controls were performed on blood donors, in whom the presence of IgA was not verified (but sIgA is rare in the general population and is reported to be around 1:600 in Caucasians) [23]. In both our study and that of Petty et al. [24], anti-IgA IgG was found even in patients with detectable serum IgA. Thus, it seems that immunisation against IgA is possible in healthy individuals, and in PID patients, even if they have no IgA deficiency. As we found low anti-IgA IgG levels in some IgP products, we cannot exclude that the anti-IgA IgG contained in IgP contribute to those detected in the patients’blood in the days or months after an IgP infusion. However, anti-IgA IgG levels in these patients were higher than in healthy controls, and amongst the 6 anti-IgA IgG-positive subjects we described in our cohort, 3 of them never received IgP, so it is clear that their anti-IgA IgG are endogenous.

### Our Work Also Shows that the Clinical Significance of Anti-IgA IgG Is Highly Controversial and Depends on the Assay Technique, as Highlighted by Others[6, 25]

The presence of anti-IgA antibodies seems to be a poor predictor of IgP-HS, for diverse reasons:

(i) The presence of anti-IgA antibodies in a patient was neither sufficient nor essential to cause adverse reactions: in the literature, we identified several patients with anti-IgA Abs without IgP-HS, and patients with IgP-HS without anti-IgA Ab.
(ii) Moreover, we did not identify specific patterns of patients with anti-IgA Abs who could be at risk of IgP-HS. Indeed, no association was found between the presence of IgP-HS and the anti-IgA Ab level, nor with the content in IgA in the IgP.

Furthermore, the results of a passive transfer experiment were not in favour of a pathogenicity of anti-IgA Abs. In that study, Robitaille et al. transferred anti-IgA-containing blood products to IgA-positive patients, and found no evidence for an increased risk of HS compared to anti-IgA-free blood products [26]. However, as we found anti-IgA IgG at low levels in some IgPs, we cannot exclude that they could play a role in some cases of IgP-HS.

In our case series, all three patients who tested positive for anti-IgA IgG and received IgRT, experienced IgP-HS (Table 2). This could argue in favour of a greater specificity of our anti-IgA IgG assay, which uses a standardised fluorescent enzyme immunoassay with a well-defined positivity threshold, unlike the various “in-house” techniques used in the literature. However, two of these three patients tolerated subsequent infusions of other IgP, with similar IgA content. Moreover, we did not test our patients for anti-IgA IgE, and cannot rule out a participation of their possible presence in the patients’ symptoms, even if the course of IgP-HS is not in line with an allergic mechanism.

Thus, even if it remains controversial, we do not think that a systematic assay of anti-IgA Abs is of great interest in PID patients, as its absence is not predictive of the absence of HS risk, and its presence is not a contraindication to the use of IgPs (regardless of whether they contain low or high levels of IgA).

### We Also Found that IgP Infusion Can Activate the Complement Cascade, Suggesting a Possible Role in IgP-HS

We assessed the possibility that some of these HS reactions could be due to a recently described HS mechanism. In this CARPA mechanism, the classical or the alternative complement cascade is directly activated by the drug (respectively, by the generation of immune complexes, or by their homology with pathogenic viruses, such as with liposomal drugs). Then, the anaphylatoxins (C3a, C5a) that are generated by the complement cascade activate the allergy-mediator secretory cells (mast cells, basophils and tissue macrophages), resulting in the secretion of numerous highly effective vasoactive inflammatory mediators. These mediators then trigger the effector cells (endothelial cells, smooth muscle cells) inducing various responses (capillary leakage, bronchoconstriction, vasodilation, etc.) [14].

The hypothesis of complement activation by IgPs has long been raised in the literature. Yet, the data are inconsistent. Several studies found a complement activation (according to CH50, total C3 or C3a levels) and an elevation of circulating immune complexes after an IgP infusion [13, 27, 28], but these results were not confirmed by others [29]. Here, we provide data which suggest that IgPs were able to activate complement, both in vivo or in vitro. The elevation of the complement split product Bb could indicate an activation of the alternative pathway; it would be interesting to complete our results by an AH50 assay to confirm this point.

This raises the question of the clinical consequence of this complement activation. Indeed, some studies have shown an increased complement activation in patients with IgP-HS [13], although others found complement activation even in patients who well tolerated IgPs [9, 27]. In our in vitro experiments, the magnitude of the complement activation was not predictive of IgP-HS, as sC5b9 fold change was not different whether the serum came from a patient who tolerated IgP well or from a patient who tolerated it poorly. This is in line with studies about CARPA with other drugs, in which the in vitro assays were useful to predict the complement activation potential of a drug, but not to predict the individual risk of a patient having an infusion reaction [30, 31]. In our in vivo experiments, all the patients presented signs of complement activation after IgP infusion, regardless of whether they presented IgP-HS. We could only assess three patients with IgP-HS. The patient with the most severe reaction presented the highest levels of complement activation split products.

It could well be that the risk of IgP-HS is not strictly proportional to the complement activation level, but rather to the speed of anaphylatoxin clearance, and to the susceptibility of mast cells to release vasoactive mediators [32]. Indeed, Chanan-Khan et al. demonstrated that with the first IV infusion of pegylated liposomal doxorubicin, a drug frequently responsible for CARPA, the higher the dose rate, the higher the sC5b9 levels, and that HS reactions were more frequent with the higher rates of infusion [32]. It should be noted that our samples for sC5b9 assessment after infusion were collected in the hour following the infusion: the peak of plasma sC5b9 may occur very early during the infusion, and it is likely that we did not obtain the maximum level of sC5b9 in our patients with a single sample [32]. Moreover, it is important to acknowledge that we could only include a small number of patients in our in vitro and in vivo experiment, and that all the PID patients had been receiving IgP for months or years: a prospective study would be warranted in a larger cohort of patients to assess (i) if complement activation is greater at the start of IgRT, (ii) if a greater complement activation is associated with more severe and/or more frequent HS symptoms, and (iii) if complement activation decreases with time, as IgP-HS decreases with time in PID patients.

The link between anti-IgA Abs and complement activation also needs to be clarified. Indeed, in theory, the presence of anti-IgA Abs could promote complement activation by the formation of immune complex with IgA-containing IgP. In the study by Cunningham-Rundles et al*.*, all the patients showed signs of complement activation with an increase in the C3a levels during the Ig infusion, whether they had anti-IgA Abs or not [28]. We could not assess the in vivo complement activation in anti-IgA IgG-positive patients in our study, but we found no difference in in vitro complement activation between patients with and those without anti-IgA IgG.

Finally, it is important to underline that some IgPs (such as Clairyg®, Gammagard®, Gammanorm®, Hizentra® and Cutaquig®) contain polysorbate 80 [33]. This compound, a derivative of polyethylene glycol, is known to be implicated in complement activation [34] and in CARPA reactions [35], as well as in IgE-mediated HS [36]. One can thus assume that this vehicle could also be implicated in some IgP-HS, and that complement activation tests and skin tests should be performed in these patients.

Overall, our results suggest that IgPs can activate the complement cascade, but further studies will be needed to clarify the clinical consequences of this complement activation.

It is important to underline that the infusion rate and the route could also contribute to the tolerance of IgP, and that some methods have been proposed to improve it. It is usually recommended to reduce the infusion speed [18, 37], to use simple analgesics if appropriate, to ensure adequate hydration, and to consider SC rather than the IV route if symptoms persist [10, 38]. Premedication with anti-histamine drugs and steroids can also be considered. An intentionally repeated exposure to IgP at short time intervals has also been proposed as a desensitisation way to improve IgP tolerance [18, 20–22].

In conclusion, our data and the findings of the literature review do not support a predictive value of anti-IgA Abs in IgP-HS. Breaking the myth of “IgA allergy” has major implications for PID patient care. Indeed, despite well-conducted IgRT, some patients with CVID or X-linked agammaglobulinaemia patients continue to experience recurrent or chronic bacterial respiratory tract infections, which may worsen bronchiectasis [37, 39]. One hypothesis to explain this could be the lack of IgA and IgM in conventional IgPs. In support of this, lower levels of IgM and IgA were associated with bronchiectasis in CVID patients [40], and low levels of IgA were associated with a higher risk of pulmonary and sinus infections [39]. These findings can support the use of IgA/IgM-enriched IgPs in selected severe PID patients [37, 41]. Our study and the high similarities with “CARPA” also suggest a role of complement activation, which will need to be confirmed in larger prospective studies in PID patients starting IgRT.

## Supplementary Information

Below is the link to the electronic supplementary material.

Supplementary file1 (DOCX 239 KB)

## Data Availability

The datasets generated during the current study are available from the corresponding author on reasonable request. The data will be provided after its de-identification, in compliance with applicable privacy laws, data protection, and requirements for consent and anonymization.
