# Supplementary material for: Non-allergic Hypersensitivity Reactions to Immunoglobulin Preparations in Antibody Deficiencies: What Role for Anti-IgA IgG and Complement Activation?
Source: Clin Rev Allergy Immunol. 2024 Oct 22;67(1-3):47–57. doi: 10.1007/s12016-024-09007-0 (PMC11638320; doi:10.1007/s12016-024-09007-0)
Supplement: Supplementary file 1 — Supplementary file1 (DOCX 239 KB) [file 12016_2024_9007_MOESM1_ESM.docx]

**Supplementary data**

**Supplementary Methods**

## *IgA and IgG immunoglobulins measures*

The IgG and IgA levels were measured on serum samples by a nephelemetric assay (BN2, Siemens).

## *In vitro complement activation assays*

As described for other drugs (1), a conventional IgP (Clairyg®) or a control solution (PBS) was incubated with the patient’s serum in a water bath for 30 min at 37°C, at an Ig to serum ratio of 1:5. More precisely, 5 μL of the IgP was added to 25 μL of serum placed in Eppendorf tubes, to generate complement activation. The reaction was stopped by adding 1000 μL of PBS containing 2 mM EDTA. One hundred microliters of this final solution was then used to measure the IgP-induced rise of the complement split product sC5b9 by an enzyme-linked immunosorbent assay (Microvue, Quidel®).

## *In vivo complement activation assays*

EDTA-plasma samples were used for all the complement analyses, as described by Lopez et al., 2019 (2). Patients were sampled just before and just after the Ig infusion (within 1 hour following the infusion). To assess CP50 activity, we used the Optilite CH50 Kit® liposome-based immunoassay. The Optilite C3c Kit® and C4 Kit® turbidimetric assays were used to measure C3c and C4 fractions. The complement split products sC5b9 and Bb were evaluated using an enzyme-linked immunosorbent assay (Microvue, Quidel®).

*Statistics*

For the in vitro complement activation tests, comparisons of sC5b9 levels after incubation with PBS and IgP were made using a paired Wilcoxon test. sC5b9 fold changes between patients with and those without IgP-HS and between patients with and those without anti-IgA IgG were compared using a Mann-Whitney test. For in vivo complement assessment, complement levels before and after the IgP infusion were compared using a paired t-test. The statistical significance threshold was fixed at 0.05.

**Supplementary Figure:**


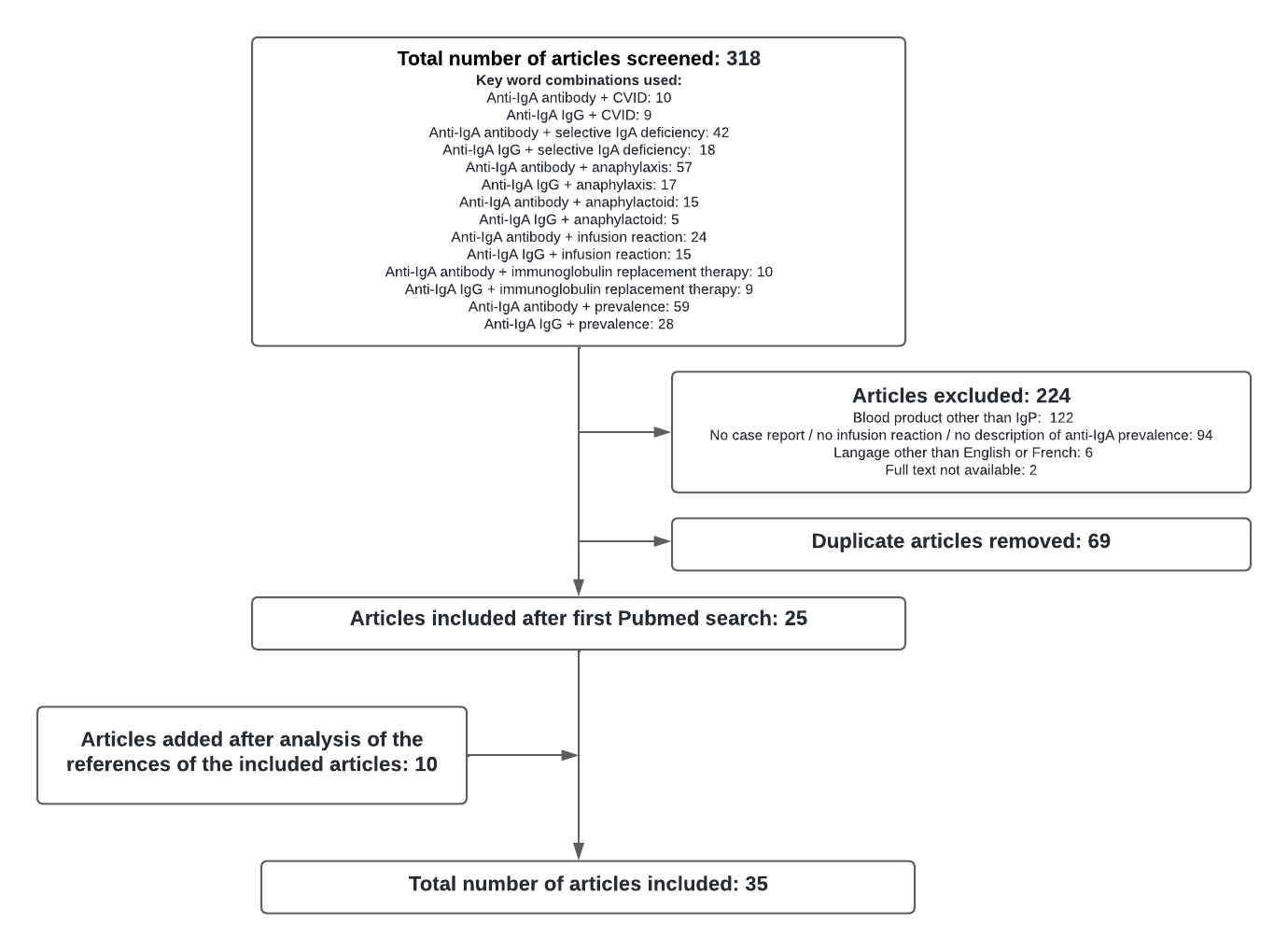


**Figure S1. Flow chart of the systematic literature review on anti-IgA antibodies in primary immunodeficiency**

Abbreviation: CVID, common variable immunodeficiency

**Supplementary Tables**

**Table S1. Prevalence of anti-IgA IgG in CVID patients**

| **Author and year** | **Number of CVID subjects** | **Anti-IgA IgG prevalence** | **Method** | **Threshold** | **Isotype** | **Specificity** |
| --- | --- | --- | --- | --- | --- | --- |
| Björkander et al., 1987 (3) | 85 | 17% | PHA then ELISA | ND | 34 IgG  3 IgM  5 IgD | ND |
| Ferreira et al., 1988 (4) | 40 | 17.5% | ELISA | Mean + 3DS | IgG | 100% subclass specific |
| Ferreira et al., 1989 (5) | 22 | 27% | ELISA | Mean + 3DS | IgG | 100% subclass specific |
| de Albuquerque Campos et al., 2000 (6) | 20 | 25% | ELISA | Mean + 3DS | IgG | ND |
| Salama et al., 2001 (7) | 21 | 19% | PGI | 1:2 dilution | ND | ND |
| Eijkhout et al., 2003 (8) | 15 | 73% | PHA | ND | ND | ND |
| Horn et al., 2006 (9) | 88 | 9% | ELISA | ND | IgG | ND |
| Torabi-Sagvand et al., 2014 (10) | 33 | 18% | ELISA | 3.6 U/mL | IgG | ND |
| Our study | 32 | 6.2% | FEIA | 10 U/mL | IgG | ND |

Abbreviations: CVID, common variable immunodeficiency; ELISA, enzyme-linked immunosorbent assay; FEIA, fluorescent enzyme immunoassay; PGI, particle gel immunoassay; PHA, passive hemagglutination assay; ND, not determined.

**Table S2. Prevalence of anti-IgA IgG in sIgA patients**

| **Author and year** | **Number of sIgA subjects** | **Anti-IgA IgG prevalence** | **Method** | **Threshold** | **Isotype** | **Specificity** |
| --- | --- | --- | --- | --- | --- | --- |
| Nadorp et al., 1972 (11) | 16 | 44% | RIA | ND | IgG | 85% class specific  15% subclass specific |
| Petty et al., 1979 (12) | 83 | 25% | PHA | 1:4 | ND | ND |
| Hammarström et al., 1983 (13) | 42 | 14% | PHA then ELISA | ND | IgG | ND |
| Björkander et al., 1987 (3) | 185 | 29% | PHA then ELISA | ND | 34 IgG  3 IgM  5 IgD | ND |
| Ferreira et al., 1988 (4) | 46 | 39% | ELISA | Mean + 3DS | IgG  1 IgG + IgE | 4 class specific  14 subclass specific |
| Sandler et al., 1994 (14) | 438 | 37% | PHA | ND | ND | 96% class specific  4% subclass specific |
| Munks et al., 1998 (15) | 357 | 28% | PHA | ND | ND | ND |
| Sundin et al., 1998 (16) | 194 | 22% | PHA then ELISA | OD > 0.4 | ND | ND |
| Salama et al., 2001 (7) | 14 | 42% | PGI | 1:2 dilution | ND | NA |
| Our study | 10 | 30% | FEIA | 10 U/mL | IgG | ND |

Abbreviations: sIgA, selective IgA deficiency; ELISA, enzyme-linked immunosorbent assay; FEIA, fluorescent enzyme immunoassay; OD, optical density; PGI, particle gel immunoassay; PHA, passive haemagglutination assay; NA, not applicable; ND, not determined; RIA, radioimmuno assay.

**Table S3. Prevalence of anti-IgA IgG in healthy subjects**

| **Author and year** | **Number of healthy subjects** | **Healthy subject category** | **Anti-IgA IgG prevalence** | **Method** | **Threshold** | **Isotype** | **Specificity** |
| --- | --- | --- | --- | --- | --- | --- | --- |
| Ropars et al., 1974 (17) | 73 | Blood donors | 0% | PHA | 1:8 dilution | NA | NA |
| Rivat et al., 1977 (18) | 1010 | Blood donors | 59% | PHA | 1:2 dilution | NA | 100% subclass specific |
| Petty et al., 1985 (19) | 200 | IgA + blood donors | 6% | PHA then ELISA | 1:4 dilution  Mean + 2DS | IgG | 5.5% subclass specific  0.5% class specific |
| Petty et al., 1985 (19) | 120 | IgA + pregnant women | 14.2% | PHA then ELISA | 1:4 dilution  Mean + 2DS | IgG | 11.7% subclass specific  2.5% class specific |
| Ferreira et al., 1988 (4) | 106 | Blood donors | 5.6% | ELISA | Mean + 3DS | IgG  1 IgM  No IgE | 20% class specific  80% subclass specific |
| de Albuquerque Campos et al., 2000 (6) | 30 | IgA+ healthy individuals | 0% | ELISA | Mean + 3DS | NA | NA |
| Salama et al., 2001 (7) | 105 | Blood donors | 0% | PGI | 1:2 dilution | NA | NA |
| Our study | 46 | Healthy individuals | 2% | FEIA | 10 U/mL | IgG | ND |

Abbreviations: ELISA, enzyme-linked immunosorbent assay; FEIA, fluorescent enzyme immune assay; PGI: particle gel immunoassay; PHA, passive haemagglutination assay; NA, not applicable; ND, not determined

**Table S4. Anti-IgA IgG levels in IgPs**

| **IgP** | **Dilution** | **Anti-IgA IgG**  N < 10 U/mL | **Interpretation** |
| --- | --- | --- | --- |
| Clairyg lot A | 1/5 | 14 | **Positive** |
| Clairyg lot A | Pure | 65 | **Positive** |
| Clairyg lot B | 1/5 | 9,3 | Equivocal |
| Clairyg lot B | Pure | 47 | **Positive** |
| Flebogamma lot A | 1/10 | 2,1 | Negative |
| Flebogamma lot A | Pure | 9,7 | Equivocal |
| Flebogamma lot B | 1/10 | 2,3 | Negative |
| Flebogamma lot B | Pure | 9,5 | Equivocal |
| IgVena | 1/10 | 3,6 | Negative |
| IgVena | Pure | 15 | **Positive** |
| Intratect | 1/5 | 17 | **Positive** |
| Intratect | Pure | 78 | **Positive** |

Abbreviations: IgP: immunoglobulins preparation

**Table S5. Description of IgP-HS symptoms in anti-IgA IgG-positive patients**

| **Author / year** | **Gender / Age (years)** | **Main diagnosis** | **IgA (g/L)** | **Anti IgA-Ab rate and isotype** | **IgP, IgA quantity, administration route** | **Type of IgP-HS** | **Number of Ig courses before reaction** | **Reaction delay after perfusion beginning** | **Tolerance of the following Ig courses** |
| --- | --- | --- | --- | --- | --- | --- | --- | --- | --- |
| Rachid et al., 2011 (20) | F/26 | CVID | ND | 3946 ng/mL, IgG | ND, ND, IV | Anaphylaxis | ND | ND | ND |
| Salama et al., 2004 (21) | M/40 | CVID | < 0.06 | 1:32, IgG | OCTAGAM  < 100 μg/mL  IV | Urticaria  Vomiting  Dyspnoea | None | A few minutes | Good tolerance |
| Ropars et al., 1974 (17) | NR | Primary ID | ND | ND, ND | ND, ND, IV | Anaphylaxis | ND | ND | ND |
| Ahrens et al., 2007 (22) | F/33 | CVID | <0.05 | 1:4, ND | INTRAGLOBIN, 2500 μg/mL, IV | Anaphylaxis | ND | ND | Good tolerance of low-speed Ig perfusion |
| Ahrens et al., 2007 (22) | F/52 | CVID | <0.05 | 1:64, ND | VENIMMUN, < 100 μg/mL, IV | Anaphylaxis | ND | ND | Good tolerance of low-speed Ig perfusion |
| Ahrens et al., 2007 (22) | M/70 | CVID | <0.05 | 1:2, ND | INTRATECT, < 2000 μg/mL, IV | Anaphylaxis | ND | ND | Good tolerance of low-speed Ig perfusion |
| Ahrens et al., 2007 (22) | F/62 | CVID | <0.05 | 1:4, ND | INTRAGLOBIN, 2500 μg/mL, IV  SANDOGLOBULIN, < 15 μg/mL, IV  ENDOBULIN, 30 μg/mL, IV | Anaphylaxis | ND | ND | Good tolerance of low-speed Ig perfusion |
| Horn et al., 2006 (9) | F/49 | CVID | <0.0009 | 1:400, IgG | OCTAGAM,< 100 μg/mL, IV  SANDOGLOBULIN, < 15 μg/mL, IV  GAMMAVENIN, No Fc part of Ig, IV | Hypotension, flush, pruritus, dyspnoea | ND | Immediate | Good tolerance of SC Ig |
| Horn et al., 2006 (9) | F/33 | CVID | <0.0009 | 1:1600, IgG | INTRAGLOBIN,2500 μg/mL, IV | Hypotension, cyanosis, oedema | ND | ND | Good tolerance of SC Ig |
| Horn et al., 2006 (9) | F/36 | CVID | <0.0009 | 1:400, IgG | ENDOBULIN,30 μg/mL, IV | Dyspnoea, fever, chills | Soon after start | ND | Good tolerance of SC Ig |
| Horn et al., 2006 (9) | M/28 | CVID | <0.0009 | 1:1640, IgG | OCTAGAM, < 100 μg/mL, IV | Capillary leak syndrome, acute renal failure, lung oedema | ND | ND | Good tolerance of POLYGLOBIN |
| Horn et al., 2006 (9) | F/33 | CVID | <0.0009 | 1:6400, IgG | OCTAGAM, < 100 μg/mL, IV | Exanthema, enanthema, respiratory tract oedema | ND | A few minutes | Good tolerance of SC Ig |
| Eijkhout et al., 2003 (8) | M/NR | CVID | <0.001 | ND, ND | ND, 600 μg/mL, IM | Anaphylactoid reaction | ND | ND | Good tolerance of SC Ig |
| Eijkhout et al., 2003 (8) | M/NR | CVID | <0.001 | ND, ND | ND, 600 μg/mL, IM | Anaphylactoid reaction | ND | ND | Good tolerance of SC Ig |
| Eijkhout et al., 2003 (8) | F/NR | CVID | <0.001 | ND, ND | ND, 70 μg/mL, IV | Anaphylactoid reaction | ND | ND | Good tolerance of SC Ig |
| Burks et al., 1986 (23) | M/15 | CVID | 0 | 1:8000, IgG and IgE | GAMMONATIV, 15 μg/mL, IV | Anaphylaxis with cardiac arrest | 4 | ND | Good tolerance of GAMMAGARD |
| de Albuquerque Campos et al., 2000 (6) | F/NR | CVID | ND | 1:3200, IgG | SANDOGLOBULI, < 15 μg/mL, IV | Malaise, dyspnoea, chest tightness | None | ND | Good tolerance of the same preparation |
| de Albuquerque Campos et al., 2000 (6) | M/NR | CVID | ND | 1:50, IgG | SANDOGLOBULIN, < 15 μg/mL, IV | Malaise | 1 | ND | Good tolerance of the same preparation |
| Vyas et al., 1968 (24) | F/18 | NR | 0 | >1000, ND | ND, ND, IM | Anaphylaxis | ND | ND | ND |
| Cunningham-Rundles et al., 1986 (25) | F/54 | CVID | <0.01 | 1:7000, IgG | SANDOGLOBULIN, < 15 μg/mL, IV | Abdominal and chest pain, myalgias, dyspnoea, fever | ND | ND | Good tolerance of GAMONATIVE  Mild reaction with GAMMAGARD |
| Cunningham-Rundles et al., 1993 (26) | M/46 | CVID | <0.01 | 1:500, IgG | SANDOGLOBULIN, < 15 μg/mL, IV | Hypotension, cyanosis, dyspnoea, abdominal and chest pain | None | ND | Mild reaction with GAMMAGARD |
| Cunningham-Rundles et al., 1993 (26) | F/27 | CVID | <0.01 | 1:200, IgG | GAMIMMUNE, 270 μg/mL  SANDOGLOBULIN, < 15 μg/mL, IV | Hypotension, myalgias, dyspnoea | 8 | ND | Good tolerance of GAMMAGARD |
| Cunningham-Rundles et al., 1993 (26) | F/54 | CVID | 0.07 | 1:500, IgG | SANDOGLOBULIN, < 15 μg/mL, IV | Myalgias, chills, fever | None | ND | Good tolerance of GAMMAGARD |
| Björkander et al., 1985 (27) | NR | CVID | <0.05 | 1:64, IgG | GAMMONATIV, < 2000 μg/mL, IV | Near fatal reaction | ND | ND | ND |
| Björkander et al., 1985 (27) | NR | CVID | <0.05 | 1:256, IgG | GAMMONATIV, < 2000 μg/mL, IV | Slight headache, nausea | ND | ND | Good tolerance of the next perfusions of GAMMONATIV |
| Björkander et al., 1985 (27) | NR | CVID | <0.05 | ND, IgG | GAMMONATIV, < 2000 μg/mL, IV | Slight headache, nausea | ND | ND | Good tolerance of the next perfusions of GAMMONATIV |
| Ferreira et al., 1988 (4) | F/20 | CVID | 0.12 | 1:12800 IgG, 0.7 ng/mL IgE | ENDOBULIN, 20 μg/mL, IV | Headache, abdominal pain, chills, circulatory collapse, dyspnoea | Many IgIM | ND | ND |
| Kamme et al., 1975 (28) | M/33 | Hypogammaglobulinaemia | <0.1 | 1:640, IgG | KABI, ND, IM | Chest pain, dyspnoea | 24 | A few seconds | Good tolerance of the next perfusions of KABI |
| Day et al., 1984 (29) | F/44 | CVID | ND | ND, IgG | ND, ND, IV | Chest and abdominal pain, vomiting | ND | 30 min | ND |
| Yocum et al., 1991 (30) | F/27 | CVID | <0.04 | ND, IgG | ND, ND, IV | Dyspnoea, chills | None | ND | Good tolerance of IgIM and GAMMAGARD |
| Hedderich et al., 1985 (31) | M/40 | CVID | 0 | 1:10240, ND | SANDOGLOBULIN, < 15 μg/mL, IV | Bronchospasm, shock | 4 | A few minutes | Good tolerance of IgA-depleted Ig |
| Liebermann et al., 2013 (32) | F/NR | CVID | ND | ND, ND | ND, < 10 μg/mL, IV | Anaphylaxis | Many IgIV | ND | Good tolerance of IgSC |
| Torabi Sagvand et al., 2014 (10) | NR | CVID | < 0.06 | > 3.6 U/L, IgG | INTRATECT, < 2000 μg/mL, IV | Mild adverse reaction | ND | ND | ND |
| Mc Cluskey et al., 1990 (33) | M/45 | Hypogammaglobulinaemia | ND | ND, ND | ND, ND, IV | Anaphylactoid reaction | ND | ND | ND |
| Seligmann et al., 1991 (34) | F/26 | Hypogammaglobulinaemia | < 0.05 | ND, IgG | ND, ND, IM | Mild and severe adverse reactions | ND | ND | ND |
| Our cohort | F/51 | sIgA | < 0.07 | 166-328 U/L, IgG | CLAIRYG, < 22 μg/mL, IV | Headache and chills | None | ND | No other perfusion |
| Our cohort | M/46 | CVID | < 0.07 | 66-131 U/L, IgG | TEGELINE, 850 μg/mL, IV | Malaise and fever at 1st perfusion  Abdominal pain + throat oedema at 2nd infusion | None | < 5 minutes | Good tolerance of GAMMAGARD and HIZENTRA |
| Our cohort | M/53 | CVID | < 0.07 | 232 U/L, IgG | CLAIRYG, < 22 μg/mL, IV | Chills, throat oedema at 1st infusion | None | ND | Good tolerance of PRIVIGEN |

Abbreviations: Ab, antibody; CVID, common variable immunodeficiency; ID, immunodeficiency; IgP, immunoglobulin preparation; IgP-HS, hypersensitivity to immunoglobulin preparation; IM, intramuscular; IV, intravenous; ND, not determined; NR, not reported; SC, subcutaneous; sIgA, selective IgA deficiency

**Table S6. Description of the anti-IgA IgG-positive patients without IgP-HS**

| **Author / year** | **Gender / Age (years)** | **Main diagnosis** | **IgA (g/L)** | **Anti IgA-Ab rate and isotype** | **IgP, IgA quantity and administration route** |
| --- | --- | --- | --- | --- | --- |
| Rachid et al., 2011 (20) | F/60 | CVID | ND | 3750.5 ng/mL, IgG | ND, ND, SC |
| Rachid et al., 2011 (20) | M/60 | CVID | ND | 544,5 ng/mL, IgG | ND, ND, SC |
| Salama et al., 2001 (7) | F/50 | sIgA | < 0.05 | 1:32, IgG | OCTAGAM, < 100 μg/mL, IV |
| Salama et al., 2001 (7) | F/53 | sIgA | < 0.05 | 1:32, IgG | OCTAGAM, < 100 μg/mL, IV |
| Salama et al., 2001 (7) | M/42 | CVID | < 0.05 | 1:8, IgG | OCTAGAM, < 100 μg/mL, IV |
| Salama et al., 2001 (7) | NR | CVID | < 0.05 | 1:4, IgG | OCTAGAM, < 100 μg/mL, IV |
| Salama et al., 2001 (7) | F/55 | CVID | < 0.05 | 1:2, IgG | OCTAGAM, < 100 μg/mL, IV |
| Koistinen et al., 1978 (35) | F/46 | CVID | < 0.01 | 1:2000, ND | ND, ND, IM |
| Limaye et al., 2001 (36) | F/49 | CVID | < 0.07 | 1:524000, ND | INTRAGAM P, < 18 μg/mL, IV |
| Sundin et al., 1998 (16) | NR | CVID | < 0.1 | +++, ND | GAMMABULIN, <5000 μg/mL, SC |
| Sundin et al., 1998 (16) | NR | CVID | < 0.1 | +++, ND | GAMMABULIN, <5000 μg/mL, SC |
| Sundin et al., 1998 (16) | NR | CVID | < 0.1 | +++, ND | GAMMABULIN, <5000 μg/mL, SC |
| Sundin et al., 1998 (16) | NR | sIgA | < 0.1 | +++, ND | GAMMABLOBULIN, <80 μg/mL, SC |
| Horn et al., 2006 (9) | M/40 | CVID | < 0.06 | 1:800, IgG | FLEBOGAMMA, 50-100 μg/mL , IV |
| Horn et al., 2006 (9) | M/44 | CVID | < 0.06 | 1:200, IgG | OCTAGAM, < 0.1 μg/mL, IV |
| Horn et al., 2006 (9) | F/31 | CVID | < 0.06 | 1:100, IgG | ND, ND, IV |
| Eijkhout et al., 2003 (8) | M/NR | CVID | < 0.001 | ND, ND | ND, 70 μg/mL, IV |
| Eijkhout et al., 2003 (8) | M/NR | CVID | < 0.001 | ND, ND | ND, 70 μg/mL , IV |
| Eijkhout et al., 2003 (8) | F/NR | CVID | < 0.001 | ND, ND | ND, 70 μg/mL , IV |
| Eijkhout et al., 2003 (8) | F/NR | CVID | < 0.001 | ND, ND | ND, ND, SC |
| Eijkhout et al., 2003 (8) | M/NR | CVID | < 0.001 | ND, ND | ND, ND, SC |
| Burks et al., 1986 (23) | F/14 | CVID | 0 | 1:640, IgG and IgE | GAMMONATIV, < 20 μg/mL, IV |
| de Albuquerque Campos et al., 2000 (6) | M/NR | CVID | ND | 1:200, IgG | SANDOGLOBULIN, < 15 μg/mL, IV |
| Vyas et al., 1968 (24) | NR | ND | 0 | 1:32, ND | ND, ND, IM |
| Vyas et al., 1968 (24) | NR | ND | 0 | 1:256, ND | ND, ND, IM |
| Björkander et al., 1985 (27) | NR | CVID | < 0.05 | ND, IgG | GAMMONATIV, < 20 μg/mL, IV |
| Björkander et al., 1985 (27) | NR | CVID | < 0.05 | ND, IgG | GAMMONATIV, < 20 μg/mL, IV |
| Björkander et al., 1985 (27) | NR | CVID | < 0.05 | ND, IgG | GAMMONATIV, < 20 μg/mL, IV |
| Björkander et al., 1985 (27) | NR | CVID | < 0.05 | ND, IgG | GAMMONATIV, < 20 μg/mL, IV |
| Björkander et al., 1985 (27) | NR | sIgA | < 0.05 | ND, IgG | GAMMONATIV, < 20 μg/mL, IV |
| Ferreira et al., 1988 (4) | NR | Hyper-IgM syndrome | 0.57 | 1:50, IgG | ENDOBULIN, 30 μg/mL, IV |
| Ferreira et al., 1988 (4) | NR | CVID | 0.01 | 1:200, IgG | ENDOBULIN, 30 μg/mL, IV |
| Ferreira et al., 1988 (4) | NR | CVID | 1.03 | 1:600, IgG | ND, 800 - 4800 μg/mL, IM |
| Ferreira et al., 1988 (4) | NR | CVID | 0.74 | 1:10, IgG | ND, 800 - 4800 μg/mL, IM |
| Ferreira et al., 1988 (4) | NR | CVID | 2.33 | 1:10, IgG | ND, 800 - 4800 μg/mL, IM |
| Ferreira et al., 1988 (4) | NR | CVID | 0.01 | 1:50, IgG | ND, 800 - 4800 μg/mL, IM |
| Ferreira et al., 1989 (5) | NR | CVID | 0.97 | 1:20, IgG | ND, 800 - 4800 μg/mL, IM |
| Ferreira et al., 1989 (5) | NR | Hyper-IgM syndrome | 0.23 | 1.08 ng/mL, IgE | ENDOBULIN, 30 μg/mL, IV |
| Kamme et al., 1975 (28) | M/36 | Hypogammaglobulinaemia | < 0.1 | 1:160, IgG | KABI, ND, IM |
| Torabi-Sagvand et al., 2014 (10) | NR | CVID | < 0.006 | > 3.6 U/mL, IgG | INTRATECT, < 2000 μg/mL, IV |
| Torabi-Sagvand et al., 2014 (10) | NR | CVID | < 0.006 | > 3.6 U/mL, IgG | INTRATECT, < 2000 μg/mL, IV |
| Torabi-Sagvand et al., 2014 (10) | NR | CVID | < 0.006 | > 3.6 U/mL, IgG | INTRATECT, < 2000 μg/mL, IV |
| Torabi-Sagvand et al., 2014 (10) | NR | CVID | < 0.006 | > 3.6 U/mL, IgG | INTRATECT, < 2000 μg/mL, IV |
| Torabi-Sagvand et al., 2014 (10) | NR | CVID | < 0.006 | > 3.6 U/mL, IgG | INTRATECT, < 2000 μg/mL, IV |
| Gilstad et al., 2002 (37) | F/40 | IgA and IgG2 deficiency | 0.0005 | ND, ND | ND, 720 μg/mL, IV |
| Ropars et al., 1974 (17) | NR | sIgA | ND | 1:8 – 1:256, ND | ND, ND, ND |
| Ropars et al., 1974 (17) | NR | sIgA | ND | 1:8 – 1:256, ND | ND, ND, ND |
| Ropars et al., 1974 (17) | NR | sIgA | ND | 1:8 – 1:256, ND | ND, ND, ND |
| Ropars et al., 1974 (17) | NR | Hypogammaglobulinaemia | ND | 1:8 – 1:256, ND | ND, ND, ND |

Abbreviations: Ab, antibody; CVID, common variable immunodeficiency; IgP-HS, hypersensitivity to immunoglobulin preparation; IM, intramuscular; IV, intravenous; ND, not determined; NR, not reported; SC, subcutaneous; sIgA, selective IgA deficiency.

**Table S7. Description of the anti-IgA IgG-negative patients with IgP-HS**

| **Gender / Age (years)** | **Main diagnosis** | **IgA (g/L)** | **IgP**  **IgA quantity**  **Administration route** | **Type of reaction** | **Number of Ig courses before IgP-HS** | **HS delay after start of perfusion** | **Tolerance of the following Ig courses** |
| --- | --- | --- | --- | --- | --- | --- | --- |
| F/41 | IgA, IgG2, IgG4 deficiency | < 0.07 | CUVITRU, 280 μg/mL, SC | Diffuse skin eruption and headache | 6 of another IgP  0 of CUVITRU | Within 24 hours | ND |
| F/27 | CVID | 0.45 | CLAIRYG, 22 μg/mL, IV  PRIVIGEN, 25 μg/mL, IV  HYQVIA, 140 μg/mL, SC  HIZENTRA, 50 μg/mL, SC  CUTAQUIG, 600 μg/mL, SC | Headache with IVIg  Headache, skin eruption and pain at injection site with SCIg | 1 perfusion of CLAIRYG | During the perfusion | Persistence of acceptable symptoms under CUTAQUIG with premedication |
| F/65 | SPAD | 0.58 | FLEBOGAMMA, 50 μg/mL, IV | Cough and dyspnoea at 2nd perfusion  Skin eruption at 3rd perfusion | Many of IGVENA  1 of FLEBOGAMMA | ND | Good tolerance of CLAIRYG |
| M/42 | CVID | 0.22 | CLAIRYG, 22 μg/mL, IV | Fever, meningeal syndrome, myalgias, nausea | 0 | A few hours | No following course |
| F/15 | Jacobsen syndrome | 0.59 | GAMMAGARD, 2.2 μg/mL, IV  CLAIRYG, 22 μg/mL, IV  GAMMANORM, 82.5 μg/mL, SC  HIZENTRA, 50 μg/mL, SC | Headache  Pain at injection site | ND | During the infusion | Persistence of headache |
| F/34 | Post-Rituximab hypogammaglobulinaemia | < 0.06 | CLAIRYG, 22 μg/mL, IV | Headache | 0 | A few hours | Good tolerance of CUTAQUIG |
| F/2 | Unspecified primitive immunodeficiency | 0.28 | CLAIRYG, 22 μg/mL, IV | Cough at 2nd perfusion  Skin eruption at the next perfusions | 1 | ND | Good tolerance with anti-histaminic premedication |
| F/35 | SPAD | 1.44 | CLAIRYG, 22 μg/mL, IV  GAMMANORM, 82.5 μg/mL, SC  PRIVIGEN, 25 μg/mL, IV | Meningeal syndrome, fever | 0 | A few hours | Good tolerance of CUVITRU |
| F/21 | CVID | <0.07 | CLAIRYG, 22 μg/mL, IV | Urticaria, oedema, chills, nausea | Many of GAMUNEX  0 of CLAIRYG | 30 minutes after the end of the infusion | Good tolerance of PRIVIGEN |
| F/43 | CVID-like | 0.13 | CLAIRYG, 22 μg/mL, IV | Chills, fever, myalgias | 0 | During the infusion | Good tolerance of CLAIRYG |

Abbreviations: CVID, common variable immunodeficiency; IgP-HS, hypersensitivity to immunoglobulin preparations; IgP, immunoglobulin preparation; IV, intravenous; ND, not determined; SC, subcutaneous; SPAD, specific polysaccharide antibody deficiency.

**Table S8. Evolution of complement activation before and after IgP infusion**

| **Gender / Age (years)** | **Main diagnosis** | **Anti-IgA IgG** | **IgP**  **IgA quantity**  **Administration route** | **IgP tolerance** |  | **C3**  N: 800-1640 mg/L | **C4**  N:120-380 mg/L | **CH50**  N: 35.40-76.30 mg/L | **C5b9**  N: 75-219 ng/mL | **Bb**  N: 0.49 - 1.42 μg/mL |
| --- | --- | --- | --- | --- | --- | --- | --- | --- | --- | --- |
| F/44 | CVID | Negative | FLEBOGAMMA  50 μg/mL, IV | Good | Before | 1253 | 174 | 58.35 | 146 | 1.86 |
|  |  |  |  |  | After | 1102 | 165 | 52.46 | 99 | 1.90 |
| M/49 | CVID | Negative | ND  ND, IV | Good | Before | 1107 | 200 | 52.37 | 157 | 0.73 |
|  |  |  |  |  | After | 980 | 167 | 46.50 | 225 | 0.98 |
| F/64 | CVID | Negative | FLEBOGAMMA  50 μg/mL, IV | Good | Before | 1246 | 320 | 62.01 | 130 | 1.38 |
|  |  |  |  |  | After | 1115 | 282 | 62.41 | 185 | 3.55 |
| F/22 | CVID | Negative | PRIVIGEN  25 μg/mL, IV | Good | Before | 1335 | 259 | 68.29 | 114 | 0.79 |
|  |  |  |  |  | After | 1248 | 249 | 63.88 | 156 | 1.07 |
| M/18 | CVID | Negative | PRIVIGEN  25 μg/mL, IV | Good | Before | 1281 | 328 | 59.64 | 62 | 0.84 |
|  |  |  |  |  | After | 1136 | 297 | 50.95 | 117 | 1.03 |
| F/58 | SPAD | Negative | CLAIRYG, 22 μg/mL, IV | Good | Before | 1196 | 226 | 47.94 | 179 | 1.08 |
|  |  |  |  |  | After | 1090 | 203 | 44.51 | 166 | 1.23 |
| F/58 | Unspecified immunodeficiency | Negative | CLAIRYG, 22 μg/mL, IV | Good | Before | 1106 | 312 | 58.33 | 104 | 0.89 |
|  |  |  |  |  | After | 933 | 274 | 50.66 | 130 | 1.46 |
| M/44 | SPAD | Negative | CLAIRYG, 22 μg/mL, IV | Good | Before | 956 | 220 | 56.75 | 109 | 1.40 |
|  |  |  |  |  | After | 921 | 211 | 52.39 | 158 | 2.01 |
| F/43 | Post-rituximab hypogammaglobulinaemia | Negative | CLAIRYG, 22 μg/mL, IV | Headache | Before | 1156 | 268 | 59.53 | 121 | 1.03 |
|  |  |  |  |  | After | 1090 | 247 | 50.35 | 170 | 1.23 |
| F/26 | CVID | Negative | PRIVIGEN  25 μg/mL, IV | Headache | Before | 1251 | 200 | 64.72 | 135 | 0.79 |
|  |  |  |  |  | After | 1199 | 199 | 54.72 | 110 | 0.80 |
| F/21 | CVID | Negative | CLAIRYG, 22 μg/mL, IV | Urticaria | Before | 1066 | 317 | 73.15 | 59 | 1.08 |
|  |  |  |  |  | After | 1084 | 326 | 70.04 | 385 | 4.31 |

Abbreviations: CVID, common variable immunodeficiency; F, female; IgP, immunoglobulin preparation; IV, intravenous; M, male; ND, not determined; SPAD, specific polysaccharide antibody deficiency.

**Supplementary References**

1. Szebeni J. Hemocompatibility testing for nanomedicines and biologicals: predictive assays for complement mediated infusion reactions. Eur J Nanomedicine [Internet]. 1 janv 2012 [cité 21 nov 2022];4(1). Disponible sur: https://www.degruyter.com/document/doi/10.1515/ejnm-2012-0002/html

2. Lopez B, Bertier N, Ledoult E, Joudinaud R, Maanaoui M, Majerus V, et al. Classical pathway activity C3c, C4 and C1-inhibitor protein reference intervals determination in EDTA plasma. Biochem Medica. 15 oct 2019;29(3):030707.

3. Björkander J, Hammarström L, Smith CI, Buckley RH, Cunningham-Rundles C, Hanson LA. Immunoglobulin prophylaxis in patients with antibody deficiency syndromes and anti-IgA antibodies. J Clin Immunol. janv 1987;7(1):8‑15.

4. Ferreira A, Garcia Rodriguez MC, Lopez-Trascasa M, Pascual Salcedo D, Fontan G. Anti-IgA antibodies in selective IgA deficiency and in primary immunodeficient patients treated with gamma-globulin. Clin Immunol Immunopathol. mai 1988;47(2):199‑207.

5. Ferreira A, García Rodriguez MC, Fontán G. Follow-up of anti-IgA antibodies in primary immunodeficient patients treated with gamma-globulin. Vox Sang. 1989;56(4):218‑22.

6. de Albuquerque Campos R, Sato MN, da Silva Duarte AJ. IgG anti-IgA subclasses in common variable immunodeficiency and association with severe adverse reactions to intravenous immunoglobulin therapy. J Clin Immunol. janv 2000;20(1):77‑82.

7. Salama A, Schwind P, Schönhage K, Genth R, Cotting C, Hustinx H, et al. Rapid detection of antibodies to immunoglobulin A molecules by using the particle gel immunoassay: Anti-IgA. Vox Sang. juill 2001;81(1):45‑8.

8. Eijkhout HW, van den Broek PJ, van der Meer JWM. Substitution therapy in immunodeficient patients with anti-IgA antibodies or severe adverse reactions to previous immunoglobulin therapy. Neth J Med. juin 2003;61(6):213‑7.

9. Horn J, Thon V, Bartonkova D, Salzer U, Warnatz K, Schlesier M, et al. Anti-IgA antibodies in common variable immunodeficiency (CVID): diagnostic workup and therapeutic strategy. Clin Immunol Orlando Fla. févr 2007;122(2):156‑62.

10. Torabi Sagvand B, Mirminachi B, Abolhassani H, Shokouhfar T, Keihanian T, Amirzargar A, et al. IgG anti-IgA antibodies in paediatric antibody-deficient patients receiving intravenous immunoglobulin. Allergol Immunopathol (Madr). août 2015;43(4):403‑8.

11. Nadorp JH, Voss M, Buys WC, van Munster PJ, van Tongeren JH, Aalberse RC, et al. The significance of the presence of anti-IgA antibodies in individuals with an IgA deficiency. Eur J Clin Invest. juill 1973;3(4):317‑23.

12. Petty RE, Palmer NR, Cassidy JT, TUBERGENt DG, Sullivan DB. The association of autoimmune diseases and anti-IgA antibodies in patients with selective IgA deficiency. :6.

13. Hammarstrom L, Persson MAA, Smith CIE. Anti-IgA in Selective IgA Deficiency.: In Vitro Effects and Ig Subclass Pattern of Human Anti-IgA. Scand J Immunol. déc 1983;18(6):509‑13.

14. Sandler S, Eckrich R, Malamut D, Mallory D. Hemagglutination assays for the diagnosis and prevention of IgA anaphylactic transfusion reactions. Blood. 15 sept 1994;84(6):2031‑5.

15. Munks R, Booth JR, Sokol RJ. A comprehensive IgA service provided by a blood transfusion center. Immunohematology. 1 janv 1998;14(4):155‑60.

16. Sundin U, Nava S, Hammarström L. Induction of unresponsiveness against IgA in IgA-deficient patients on subcutaneous immunoglobulin infusion therapy. Clin Exp Immunol. mai 1998;112(2):341‑6.

17. Ropars C, Caldera LH, Griscelli C, Homberg JC, Salmon C. Antiimmunoglobulin Antibodies in Immunodeficiencies: Their Influence on Intolerance Reactions to γ-globulin Administration. Vox Sang. oct 1974;27(4):294‑301.

18. Rivat L, Rivat C, Daveau M, Ropartz C. Comparative frequencies of anti-IgA antibodies among patients with anaphylactic transfusion reactions and among normal blood donors. Clin Immunol Immunopathol. mai 1977;7(3):340‑8.

19. Petty RE, Sherry DD, Johannson J. Anti-IgA Antibodies in Pregnancy. N Engl J Med. 26 déc 1985;313(26):1620‑5.

20. Rachid R, Castells M, Cunningham-Rundles C, Bonilla FA. Association of anti-IgA antibodies with adverse reactions to γ-globulin infusion. J Allergy Clin Immunol. juill 2011;128(1):228-230.e1.

21. Salama A, Temmesfeld B, Hippenstiel S, Kalus U, Suttorp N, Kiesewetter H. A new strategy for the prevention of IgA anaphylactic transfusion reactions. Transfusion (Paris). avr 2004;44(4):509‑11.

22. Ahrens N, Höflich C, Bombard S, Lochs H, Kiesewetter H, Salama A. Immune tolerance induction in patients with IgA anaphylactoid reactions following long-term intravenous IgG treatment. Clin Exp Immunol. mars 2008;151(3):455‑8.

23. Burks AW, Sampson HA, Buckley RH. Anaphylactic reactions after gamma globulin administration in patients with hypogammaglobulinemia. Detection of IgE antibodies to IgA. N Engl J Med. 27 févr 1986;314(9):560‑4.

24. Vyas GN, Perkins HA, Fudenberg HH. Anaphylactoid transfusion reactions associated with anti-IgA. Lancet Lond Engl. 10 août 1968;2(7563):312‑5.

25. Cunningham-Rundles C, Wong S, Björkander J, Hanson LA. Use of an IgA-depleted intravenous immunoglobulin in a patient with an anti-IgA antibody. Clin Immunol Immunopathol. févr 1986;38(2):141‑9.

26. Cunningham-Rundles C, Zhou Z, Mankarious S, Courter S. Long-term use of IgA-depleted intravenous immunoglobulin in immunodeficient subjects with anti-IgA antibodies. J Clin Immunol. juill 1993;13(4):272‑8.

27. Björkander J, Wadsworth C, Hanson LÅ. 1040 Prophylactic infusions with an unmodified intravenous immunoglobulin product causing few side-effects in patients with antibody deficiency syndromes. Infection. mai 1985;13(3):102‑10.

28. Kamme C, Dahiquist E, Jonsson S, Lindstrom F. IgG ANTIBODIES TO IgA IN TWO PATIENTS WITH HYPOGAMMAGLOBULINAEMIA TREATED WITH COMMERCIAL GAMMAGLOBULIN. Acta Pathol Microbiol Scand [C]. 15 août 2009;83C(3):189‑94.

29. Day NK, Good RA, Wahn V. Adverse reactions in selected patients following intravenous infusions of gamma globulin. Am J Med. 30 mars 1984;76(3A):25‑32.

30. Yocum MW, Kelso JM. Common Variable Immunodeficiency: The Disorder and Treatment. Mayo Clin Proc. janv 1991;66(1):83‑96.

31. Hedderich U, Kratzsch G, Stephen W, Dichtelmüller H, Olischläger K, Heimpel H. Immunoglobulin substitution therapy in a patient with primary hypogammaglobulinaemia and anti-IgA antibodies. Clin Exp Allergy. juill 1986;16(4):339‑44.

32. Lieberman P, Rachid R. Administration of Intravenous Immunoglobulin to a Patient with Hypogammaglobulinemia and Anti-IgA Antibodies. J Allergy Clin Immunol Pract. nov 2013;1(6):704.

33. Mccluskey DR, Boyd NAM. Anaphylaxis with intravenous gammaglobulin. The Lancet. oct 1990;336(8719):874.

34. Seligmann M, Aucouturier P, Danon F, Preud’Homme JL. Changes in serum immunoglobulin patterns in adults with common variable immunodeficiency. Clin Exp Immunol. 28 juin 2008;84(1):23‑7.

35. Koistinen J, Heikkilä M, Leikola J. Gammaglobulin treatment and anti-IgA antibodies in IgA-deficient patients. Br Med J. 30 sept 1978;2(6142):923‑4.

36. Limaye S, Walls RS, Riminton S. Safe and effective use of chromatographically purified intravenous immunoglobulin despite profound anti-IgA sensitization. Intern Med J. juin 2001;31(4):256‑7.

37. Gilstad CW, Kessler C, Sandler SG. Transfusing patients with anti-immunoglobulin A subclass antibodies: Letters. Vox Sang. nov 2002;83(4):363‑363.
